# Three‐dimensional microscopy and image fusion reconstruction analysis of the thyroid gland during morphogenesis

**DOI:** 10.1002/2211-5463.13150

**Published:** 2021-04-01

**Authors:** Rui‐jia Zhang, Liu Yang, Feng Sun, Ya Fang, Xiao‐ping Ye, Huai‐dong Song, Mei Dong

**Affiliations:** ^1^ Department of Molecular Diagnostics & Endocrinology The Core Laboratory in Medical Center of Clinical Research State Key Laboratory of Medical Genomics Shanghai Ninth People's Hospital Shanghai Jiao Tong University School of Medicine China

**Keywords:** Zebrafish, thyroid gland, embryonic development, 3D reconstruction, fibroblast growth factors, endoderm

## Abstract

Thyroid dysgenesis (TD) is a major cause of primary congenital hypothyroidism; however, the molecular mechanism underlying this process is unclear. Current knowledge regarding the morphogenesis of the thyroid gland and vascular anomalies affecting thyroid development is limited. To monitor the early stages of thyroid gland development, we generated double transgenic zebrafish embryos Tg(*tg*:mCherry/*flk1*:EGFP). We described the volume of the thyroid from 2 days postfertilization (dpf) to 5 dpf using 3D reconstruction images. We treated zebrafish embryos with the fibroblast growth factor (FGF) inhibitor PD166866 to better understand the impact of vascular defects on thyroid development and the effects of drug administration at specific time periods on different stages of thyroid development. The 3D reconstruction data revealed that the thyroid glands underwent significant transformation at critical time points. PD166866 treatment from 48 to 72 hours postfertilization (hpf) and from 72 to 96 hpf did not cause obvious reductions in thyroid volume but did result in observable abnormalities in thyroid morphology. The treatment also affected thyroid volume from 36 to 48 hpf, thus indicating that there are time‐point‐specific effects of drug administration during thyroid development. Three‐dimensional image reconstruction provides a comprehensive picture of thyroid anatomy and can be used to complement anatomical fluorescence information. The effects of an FGF pathway inhibitor on thyroid development were determined to be time‐point‐dependent.

## Graphical abstract

We investigated an Imaris‐based image rendering method to visualize the 3D morphology of the developing zebrafish thyroid. Using this method, we determined the thyroid volume from 2 to 5 dpf. We treated zebrafish embryos with an FGF inhibitor, and we used 3D reconstruction data to assess the effects of FGF pathway inhibitors on thyroid development at various time points.

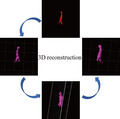

**Abbreviations**
CH: congenital hypothyroidism
dpf: days postfertilization
FGF: fibroblast growth factor
HA: hypobranchial artery
hpf: hours postfertilization
OFT: heart outflow tract
TD: thyroid dysgenesis
VA: ventral aorta

Impaired structural development of the thyroid gland is a leading cause of congenital hypothyroidism (CH) [1]. CH is one of the most frequent congenital thyroid diseases and occurs in 1 : 2000 to 1 : 3500 newborns according to worldwide neonatal screening information [2]. Nearly 85% of Caucasian newborns that are diagnosed with CH experience thyroid dysgenesis (TD) that is characterized by a complete lack of the thyroid gland (athyreosis), hemiagenesis, hypoplasia, and ectopic localization (primarily occurring in the sublingual region) [3]. Although TD accounts for the majority of CH, the pathogenic mechanisms of the molecular signal cascades underlying TD remain unclear [1, 4, 5]. According to Nilsson *et al*. [6], the thyroid follicular cells of humans and mice originate from a group of endoderm cells within the midline primordium of the pharyngeal floor. Under the control of transcription factors and other regulatory genes, thyroid progenitor cells migrate into the subpharyngeal mesenchyme and differentiate into structural and functional thyroid follicular cells. Existing data indicate that the transcription factors *NKX2‐1*, *PAX8*, and *HHEX* are essential for thyroid precursor cell specification and that the transcription factor *FOXE1* plays an important role in the process of thyroid migration [6, 7].

The thyroid gland is a highly conserved organ in vertebrates that regulates a wide range of body development processes and controls homeostatic fundamental physiological mechanisms. Zebrafish are emerging as a powerful animal model for thyroid development studies due to their external fertilization, transparent developing embryos, rapid organ development, and high interspecies conservation rates. In zebrafish, thyroid specification can be observed as early as 24 hours postfertilization (hpf) when a small number of endoderm cells begin to co‐express the three transcription factors *nkx2.4b*, *pax2a*, and *hhex* in a region closely opposed to the apical pole of the primitive heart tube [8, 9, 10]. These cells then differentiate into structural and functional thyroid follicular cells through migration along the vessels in a manner that is similar to that of the thyroid organogenesis pattern in humans [11, 12]. Furthermore, by generating zebrafish transgenic lines (e.g., the Tg(*tg*:mCherry) line) and labeling thyroid follicular cells, the development processes and dynamic changes in the thyroid can be tracked in live embryos [13]. However, there is currently no simple method for measuring and visualizing thyroid volumes in zebrafish. 2D imaging is currently the widely used method; however, it does not fully reflect thyroid morphogenesis.

Important tissue–tissue interactions have been predicted to occur between the thyroid primordium and adjacent tissues such as the cardiac mesoderm and blood vessels to guide specific morphogenic events [14]. It has been reported that the thyroid primordium is opposed to mesodermal tissue that forms the heart outflow tract (OFT) in mice and that abnormal thyroid development occurs in conjunction with defective OFT development [14, 15]. In zebrafish, it has also been reported that after budding from endoderm cells, the thyroid primordium is located in close proximity to the OFT at ~ 48 hpf [13]. After that time, the thyroid migrates along the hypobranchial arteries (HAs) and ventral aorta (VA) toward the head [13, 16]. Recently, treatment with molecular chemical compounds affecting vascular development in zebrafish has been demonstrated to inhibit thyroid migration away from the heart, ultimately leading to thyroid ectopy [17]. Further research is needed to measure and visualize the thyroid volume during normal and ectopic thyroid morphogenesis.

In this study, we crossed the Tg(*tg*:mCherry) line with a previously established transgenic reporter line Tg(*flk1*:EGFP) that expresses enhanced green fluorescent protein (EGFP) in the vascular endothelium [18]), and we generated double transgenic embryos that provided insights into the dynamics of thyroid morphogenesis in the context of the adjacent pharyngeal vasculature. Fibroblast growth factors (FGFs) and their receptors (FGFRs) stimulate neovascularization [19]. Previous studies have demonstrated that FGF signaling affects vascular development and is required for the maintenance of blood vessel integrity [20, 21, 22]. Transgenic embryos were treated with an FGF inhibitor to induce vascular anomalies in an effort to better understand the impact of vascular defects on the different stages of thyroid development. 3D imaging reconstruction was used to characterize thyroid development and the surrounding blood vessels in zebrafish in greater detail. This method can be generalized for use in other organogenesis studies in zebrafish.

## Materials and methods

### Zebrafish husbandry and embryo culture

Zebrafish maintenance was managed under standard conditions as previously described [23]. The zebrafish facility and the study protocols were approved by the Ethics Committee of Shanghai Ninth People's Hospital affiliated with the Shanghai Jiao Tong University School of Medicine (Shanghai, China). The methods were performed in accordance with the approved guidelines. Zebrafish embryos (within 5 dpf) were produced by natural spawning and raised in egg water containing 0.002% methylene blue as a fungicide in a light‐controlled incubator at 28 ± 0.5 °C. Embryos within 5 dpf possessed no sexual differentiation, and thus, no sex effect was observed in our study.

### Fish strains

The following zebrafish lines were used in this study as previously described and included Tg(*flk1*:EGFP) [18] and Tg(*tg*:mCherry) [13].

### Transgenesis

To generate transgenic animals, wild‐type (WT) embryos were co‐injected with 50 ng·mL^−1^ of Tol2(*tg*:mCherry) vector (donated by Opitz R) and 40 ng·mL^−1^ of Tol2 transposase mRNA at the one‐cell stage. Capped mRNA encoding for Tol2 transposase was generated by *in vitro* transcription using an mMachine SP6 Kit (Ambion, San Diego, CA, USA) and NotI‐linearized pCS‐zT2TP plasmid [24] as a template. Injected embryos were examined with epifluorescence microscopy for mCherry expression in thyroid tissue using a confocal microscope (Nikon A1, Tokyo, Japan). Embryos displaying mosaic mCherry expression in the thyroid region were allowed to grow to adulthood. Individual F0 founders transmitting a robust and thyroid‐specific reporter signal to their progeny were identified by outcrossing WT animals.

### FGF pathway inhibitor treatment

The FGF signaling pathway is a key pathway in thyroid specification and early thyroid organogenesis [17]. The FGF inhibitor PD166866 was dissolved in DMSO to prepare a stock solution of 20 mm. For embryo treatment, the stock solution was diluted to 1 : 5000 in embryo media. Embryos were treated from 36 to 96 hpf, 36 to 48 hpf, 48 to 72 hpf, and 72 to 96 hpf with 4 μm PD166866 and 0.02% DMSO final concentrations, where the DMSO treatment served as the vehicle control treatment. Three independent experiments were conducted. At least 20 embryos were analyzed for each experiment. All phenotypes described were consistently observed in over 70% of the embryos undergoing drug treatments.

### Image processing

The embryos were anesthetized using 0.016% tricaine (Sigma, St. Louis, MO, USA) and embedded in 1% low melting point agarose, and a confocal microscope (Nikon A1) was used to obtain photographic images. The images were acquired using a 20× objective lens and 1.5× electronic magnification. The Z‐stack step thickness was 2 μm, and the Z‐stack number was determined according to the fluorescence signal. For 48 and 72 hpf embryos, the Z‐stack number was ~ 20. For 96 and 120 hpf embryos, the Z‐stack number was ~ 25. 3D images were processed using Imaris 9.0 software (Bitplane, Zurich, Switzerland). The confocal images were opened under the Surpass interface ‘3D view’ in maximum intensity projection mode, and ‘Add cell’ was then selected. The software then recognized the fluorescence image and produced an artificial 3D photograph. By adjusting the simulated signal, the artificial photographs were matched to the fluorescence images. The volume and area were both calculated using the software. ‘Slice view’ was then selected to calculate the maximum length and width.

### Statistics

All data are presented as mean ± SEM. Student's *t*‐test was used to analyze differences between the groups. Statistical significance was set at *P* < 0.05. All analyses were conducted using graphpad prism software (version 6.0; GraphPad, San Diego, CA, USA).

## Results

### 3D simulated images illustrate a normal thyroid development process from 48 to 120 hpf

Transgenic zebrafish expressing fluorescent reporters in specific cell types, tissues, or organs provide powerful tools for phenotypic analyses of tissue morphogenesis. Thyroglobulin (tg) mRNA expression in zebrafish is induced at 32 hpf and is confined to thyroid cells [11]. By injecting embryos with the Tol2(*tg*:mCherry) plasmid, we generated a stable transgenic line that expressed the cell member‐localized red fluorescent protein mCherry exclusively in thyroid cells. The Tg(*tg*:mCherry) line exhibited early onset of reporter expression at ~ 36 hpf and restricted expression in thyroid cells as previously reported (data not shown) [13].

**Fig. 1:**
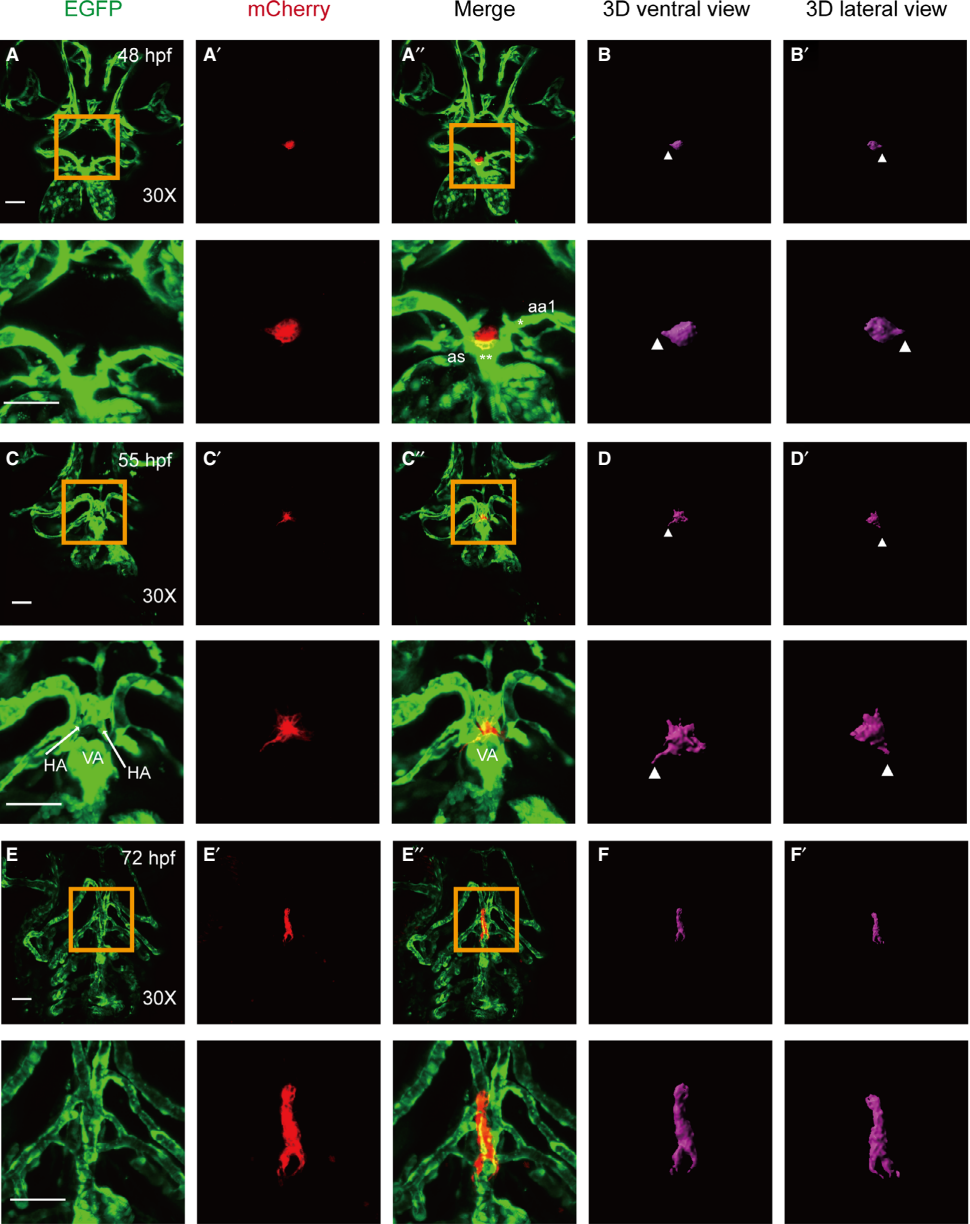
Confocal microscopy images of thyroid budding, migration, and expansion from 48 to 72 hpf with 3D simulated images. All embryos shown are oriented anterior to the top in the ventral views. (A, B) at 48 h postfertilization (hpf). The thyroid primordium is located near the OFT, is surrounded by aortic arch artery 1 (aa1), and possesses a globular appearance (A). 3D simulated images reveal that the thyroid is sphere‐shaped with a small number of small protrusions (B). (C‐D) From ~ 55 hpf, mature thyroid follicles begin to appear, and the morphology of the thyroid gland undergoes severe transformation with numerous protrusions extending in all directions, thus suggesting that the thyroid has begun to migrate. The morphology was easily recognized using the simulated 3D image. In the early development of the thyroid, an increased number of long protrusions can be observed more clearly in 3D images than they can in images produced using confocal microscopy stacking imaging techniques (B, D). (E‐F) Thyroid expansion and proliferation begin at 72 hpf along with the extension of the HAs. The thyroid extends along the HAs, and the shape of the thyroid becomes an inverted ‘Y’ when viewed in the ‘head‐up’ position (E). Three‐dimensional microscopy and image fusion reconstructions were conducted to visualize the thyroid volume (F). Magnified views of the yellow box are presented below the corresponding images. * aa1: aortic arch artery 1; ** as: aortic sac. Arrowhead: protrusions of the thyroid in 3D view. Scale bar: 50 μm.

**Fig. 2:**
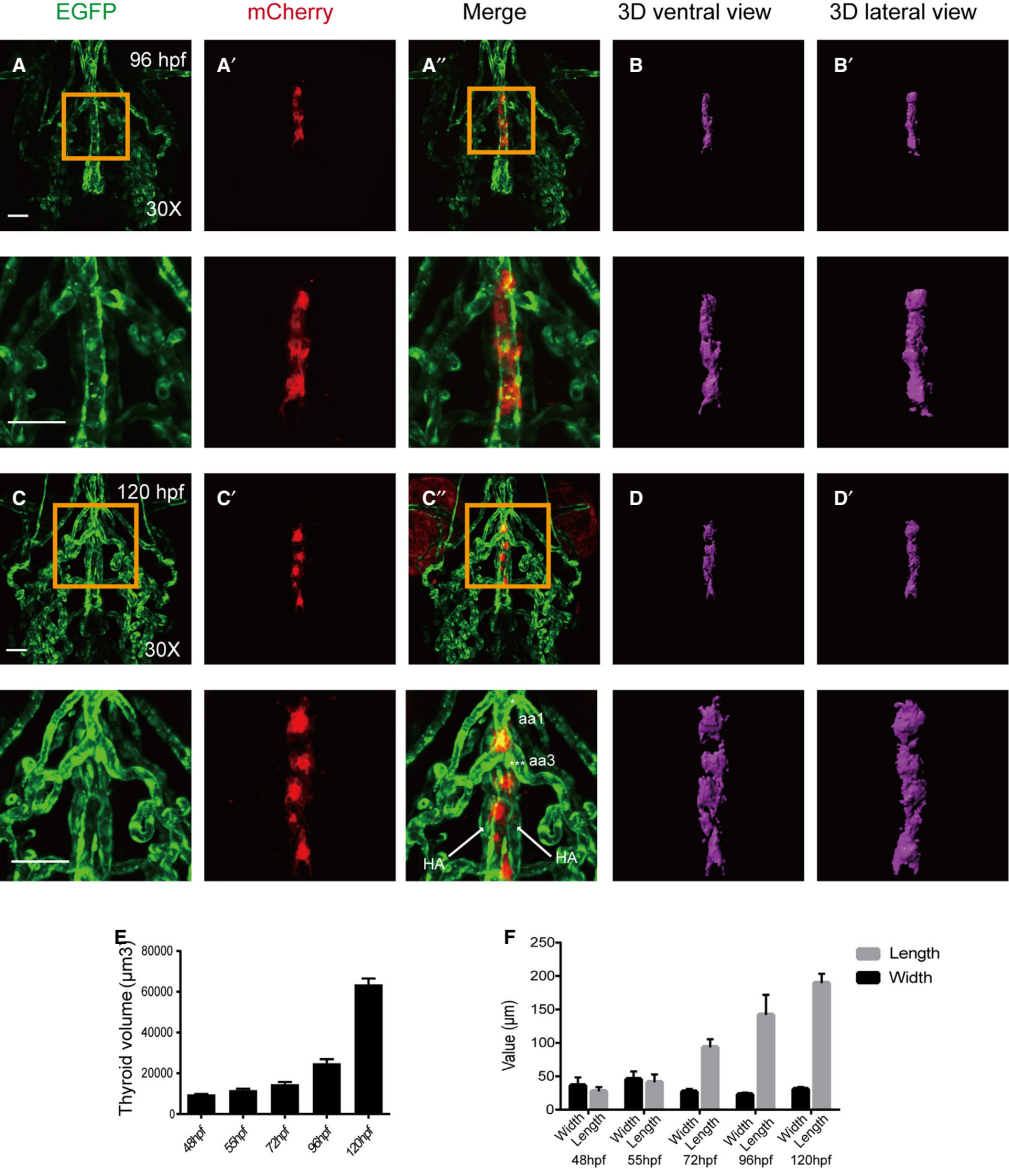
Confocal microscopy images of thyroid expansion from 96 to 120 hpf with 3D simulated images. All embryos shown are oriented anterior to the top in the ventral views. (A‐B) the thyroid is slimmer and shaped more like a long column at 96 hpf. (C‐D) Expansion of thyroid tissue moving away from the heart along the pharyngeal midline occurs at 120 hpf. (E) Thyroid volume expands gradually from 48 to 72 hpf and more drastically from 96 to 120 hpf. (F) Length increases gradually from 48 to 120 hpf, while the width remains almost constant, and this is consistent with the volume changes at the corresponding time point. Magnified views of the yellow box are presented below the corresponding images. * aa1: aortic arch artery 1; *** aa3: aortic arch artery 3. Scale bar: 50 μm. The error bars represent SEM. Data are representative of three independent experiments with similar results.

To better examine the critical early events in thyroid development that are relevant to blood vessels, double transgenic embryos (Tg[*tg*:mCherry/*flk1*:EGFP]) were introduced, where EGFP expression was used to label the vascular endothelium. Confocal live imaging was used to characterize changes in thyroid morphology from 48 to 120 hpf and to then establish a three‐dimensional atlas of normal thyroid development (Figs 1 and 2). These analyses were used to identify distinct phases of thyroid organogenesis as detailed below.

At 48 hpf, the thyroid primordium adopted a position in close proximity to the OFT, was surrounded by the aortic arch artery 1 (aa1), and exhibited a globular appearance. 3D simulated images revealed that the thyroid was sphere‐shaped and possessed a number of small protrusions (Fig. 1A,B and Video S1). At ~ 55 hpf, mature thyroid follicles began to appear, and the morphology of the thyroid gland exhibited severe transformation characteristics with numerous protrusions extending in all directions, thus suggesting that the thyroid was beginning to migrate. This morphology is more easily recognized in the simulated 3D image (Fig. 1C,D and Video S2). It has previously been reported that the HA and the VA are the primary vessels that guide thyroid migration [13, 16]. During this study, we also observed that the thyroid was surrounded by two HAs and one VA (Fig. 1C).

At 72 hpf, the thyroid extended along the paired HA, and the shape of the thyroid resembled an inverted ‘Y’ when viewed in the head‐up position. This shape was dramatically different compared with that of the previous stages (Fig. 1E,F and Video S3). At 96 hpf, the thyroid was slimmer and resembled a long column (Fig. 2A,B and Video S4). At 120 hpf, the thyroid morphology became longer compared with its size at earlier stages, and the thyroid adopted a midline position that was just anterior to the aortic arch artery 1 bifurcation (Fig. 2C,D and Video S5).

Using simulated 3D images, we analyzed the volume and area of the thyroid at each time point, and we observed that the thyroid volume expanded gradually from 48 to 72 hpf and then more drastically from 96 to 120 hpf (Fig. 2E). We also calculated the length and width of the thyroid at each stage of development, and we found that, consistent with the volume changes, the length increased gradually from 48 to 120 hpf and the width remained almost constant (Fig. 2F).

### Vessel defects affecting thyroid morphologies revealed by 3D reconstruction at different developmental stages

**Fig. 3:**
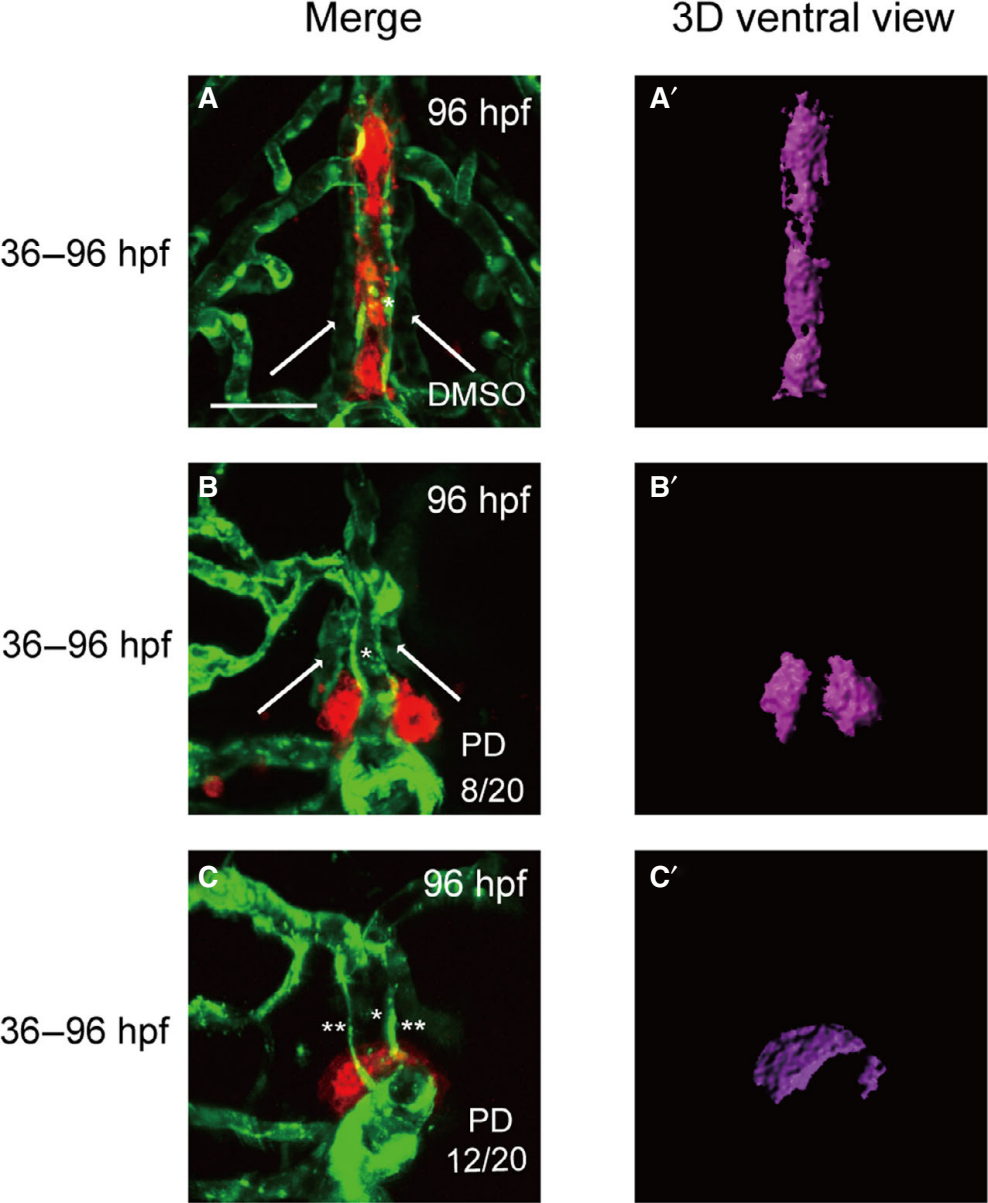
Treatment of embryos with the FGFR1‐selective inhibitor PD166866 from 36 to 96 hpf caused severe defects in thyroid morphology and volume and resulted in abnormal or absent in HAs. All embryos shown are oriented anterior to the top in the ventral views. (A) An analysis of DMSO‐treated embryos showed normal vascular development with normal thyroid morphology at 96 hpf. (B) Treatment with PD 166866 from 36 to 96 hpf caused a reduction in *tg* signal intensity and resulted in abnormal HAs (stubby and malformed) in 8 of 20 embryos. (C) The absence of HAs was observed in 12 of 20 embryos during 36–96 hpf drug treatment, and this occurred along with abnormal thyroid expansion. The percentage of abnormal or absent HAs in PD 166866‐treated embryos is presented in the lower right corner of B and C. Arrow: HA. *: VA. **: the absence of HA. Scale bar: 50 μm.

**Fig. 4:**
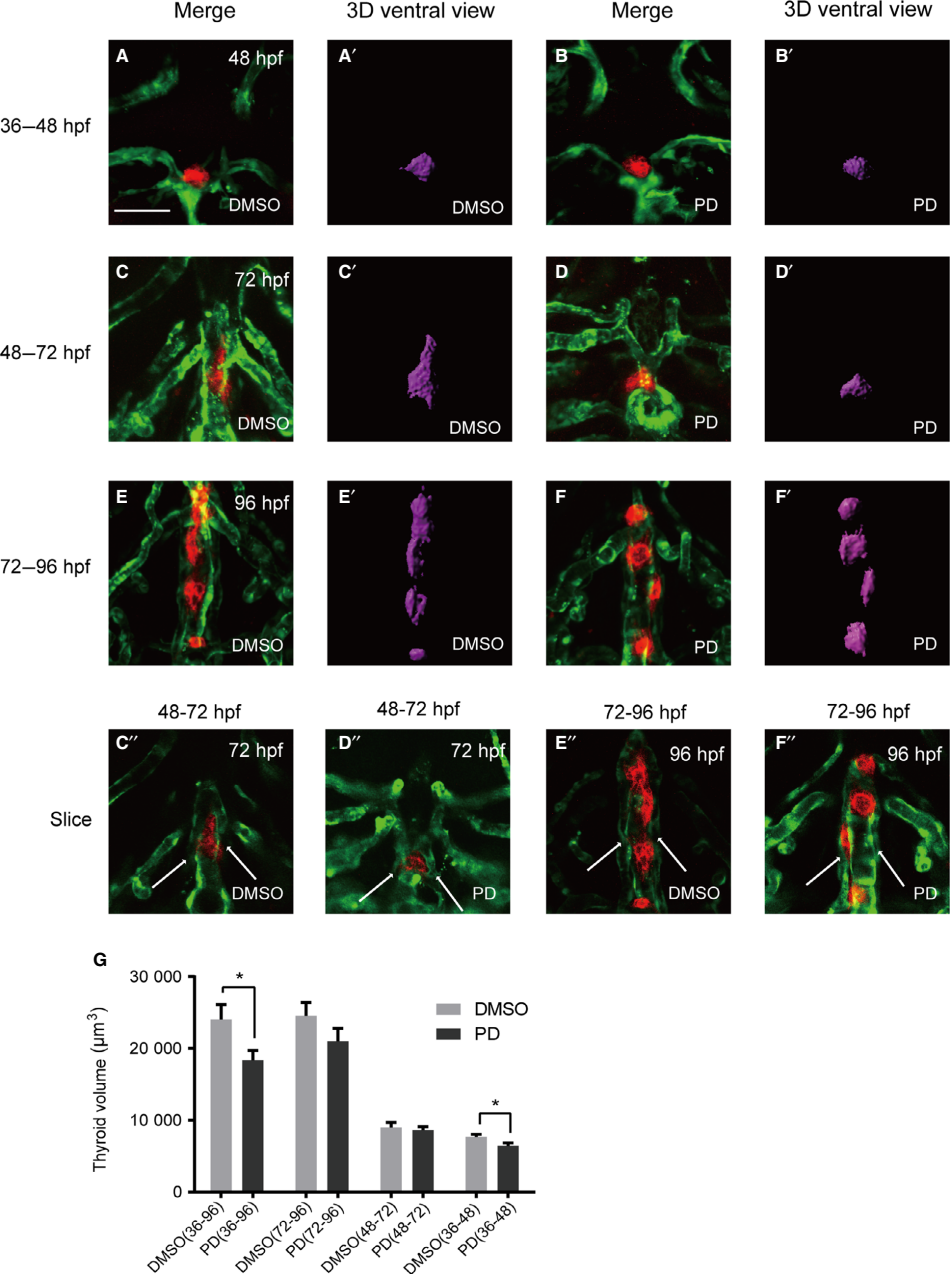
Treatment of embryos with the FGFR1‐selective inhibitor PD166866 from 36 to 96 hpf caused severe defects in thyroid morphology and volume and in HAs. All embryos shown are oriented anterior to the top in the ventral views. (A, C, E) Analysis of DMSO‐treated embryos showed normal vascular development with normal thyroid morphology. (B) Treatment with PD 166866 from 36 to 48 hpf caused a reduction in the signal intensity of *tg*. (D) During 48 to 72 hpf drug treatments, the typical inverted ‘Y’ shapes of the thyroid were barely observed in embryos after drug treatment. (F) In response to drug treatment from 72 to 96 hpf, the thyroids possessed loose and short morphology compared with those in the nondrug treatment group according to 3D simulated images, and no obvious abnormal pharyngeal vasculature surrounding the thyroid was observed. (C’’‐F’’) To better visualize HA development in embryos, the slices of C to F were presented separately. (D’’) Abnormal HAs (stubby and malformed) were observed in all embryos during 48–72 hpf drug treatment. (G) Treatment with PD166866 from 36 to 48 hpf resulted in a reduction in thyroid volume compared with that observed in vehicle‐treated embryos (*P* < 0.05). Drug treatment from 36 to 96 hpf resulted in a significant reduction in thyroid volume (*P* < 0.05). Drug treatment from 48 to 72 hpf and 72 to 96 hpf resulted in a mild effect on thyroid volume. Arrow: HA. Scale bar: 50 μm. The error bars represent SEM. Data are representative of three independent experiments with similar results. We conducted statistical comparisons using Student's *t*‐test for quantitative measures. *P < 0.05.

It has been previously reported that blood vessels play important roles in thyroid development [13, 16, 25]; however, current knowledge regarding the effect of vascular anomalies on thyroid development remains limited. We treated zebrafish embryos with an FGF inhibitor (PD166866) at different developmental stages to visualize the effect of blood vessel defects on thyroid morphology. During the period of drug treatment from 36 to 96 hpf, the embryos exhibited diverse vessel defects compared with those observed in controls (Fig. 3A‐C). The VAs were still present in embryos; however, HAs were conspicuously abnormal (stubby and malformed) (Fig. 3B) or barely detectable (Fig. 3C). Additionally, we observed that thyroid follicles did not align along the pharyngeal midline during larval growth and instead formed an irregular group around the cardiac OFT. The volume of the thyroid in the drug‐treated embryos was smaller than that of the vehicle‐treated embryos (Fig. 3B',C' and 4G). It has previously been reported that zebrafish thyroid specification and budding begin at 24 hpf and end at ~ 40 hpf [26, 27]. Different drug administration periods were introduced in our study to analyze the effect of the FGF pathway on thyroid development over time. Only the 36–48 hpf drug administration period affected thyroid volume in most of the embryos, and no obvious abnormal pharyngeal vasculature surrounding the thyroid was observed (Fig. 4A,B,G). Treatments from 48 to 72 hpf and 72 to 96 hpf did not cause obvious reductions in thyroid volume; however, they did cause observable abnormalities in thyroid morphology (Fig. 4C‐F,G). At 72 hpf, the typical inverted ‘Y’ shapes of the thyroid were barely observed in zebrafish embryos after drug treatment (Fig. 4D). Meanwhile, severe defects in pharyngeal vasculature, particularly in HAs, were observed during the 48–72 hpf drug treatments (Fig. 4D,D″). During the 72–96 hpf drug treatments, the thyroid possessed a loose and short morphology compared with that in the nondrug treatment group (Fig. 4F); however, no obvious abnormalities were observed in the VAs and HAs of the drug‐treated embryos (Fig. 4F″).

## Discussion

In this study, three‐dimensional microscopy and image fusion reconstruction were used to reveal critical time points in the stages of thyroid development. Double transgenic zebrafish embryos from the Tg(*tg*:mCherry/*flk1*:EGFP) line enabled the visualization of dynamic changes in thyroid budding, migration, proliferation, and location and provided a description of the relationship between thyroid and cardiovascular development in zebrafish. Budding of the thyroid from the pharyngeal epithelium is completed by 40 hpf, and the first follicular structures possessing established epithelial polarity are formed by 55 hpf [26]. At 48 hpf, the thyroid relocated to the aortic sac, and the thyroid primordium stacked together as tightly packed spheres (Fig. 1A,B). Our 3D simulated images revealed that the volume of the thyroid was ~ 10 000 μm^3^ in zebrafish embryos (Fig. 2E). At 55 hpf, the expansion of the thyroid tissue along the pharyngeal midline was evident in transgenic zebrafish (Fig. 1C,D). The volume of the thyroid sharply increased from 55 hpf until the end of the observation period (120 hpf), and the total increase was greater than 600% (Fig. 2E). This increase in thyroid volume resulted from an increase in length rather than a change in width (Fig. 2F) or depth (data not shown). 3D simulated images are more detailed and enable visualization of transformation during thyroid development. In the early development of the thyroid, an increased number of long protrusions can be observed more clearly in 3D simulated images than they can in images produced using confocal microscopy stacking imaging techniques (Fig. 1A–D). Previous studies estimated the thyroid volume of zebrafish by quantifying the number of *tg*‐positive cells [28, 29]. The volume of the thyroid is ~ 20 000 μm^3^ in zebrafish embryos at 48 hpf, ~ 30 000 μm^3^ at 72 hpf [29], and 60 000 μm^3^ at 120 hpf [28]. Although the volume of the thyroid at 120 hpf in the previous study was similar to that observed in this study, the volumes of thyroid at two early time points appeared to be larger than the volumes observed in this study. Previous studies used volocity software (PerkinElmer, Waltham, MA, USA) to quantify both the number and intensity of fluorescent pixels in the same area and number of stacks for each sample to define the volume of *tg*‐positive cells. In our method, we first construct 3D images based on fluorescence signals and then measured the volume. For fluorescence images, there were some spurious fluorescent signals in addition to the real signal, and we assessed the fluorescence signal fidelity prior to constructing the 3D images. As we used different software and calculation methods to estimate the volume of thyroid, it is reasonable that were observed some differences in the thyroid volume. However, the changes in thyroid volume between 48 and 72 hpf are in agreement with those of other studies. Thus, despite the use of different software and calculation methods, our study is equivalent to previous studies in regard to investigating changes in the volumes of the thyroid during the development of zebrafish embryos.

A common speculation is that the adjacent mesodermal tissues induce and permit signals for endoderm‐derived organ specification and differentiation [17, 30, 31, 32]. In zebrafish *fgf8*‐gene‐disrupted embryos, reduced thyroid primordium size at the early stages of development and a reduced number of follicles after differentiation indicate that *fgf8* is required for normal thyroid development [25]. Pharmacological approaches are advantageous with respect to temporal control in pathway modulation, as this is difficult to achieve in genetic model systems [33, 34]. Chemical genetic approaches based on small molecules provide useful tools for eliminating FGF signaling completely during specific time windows [17]. In our study, to verify the FGF pathway inhibitor effects on the development of the thyroid, another FGF inhibitor (SU5402) was administered to zebrafish at 10 μm as previously reported [17, 25]. However, ventricular defects are more marked in embryos that exhibit severe pericardial edema after 72 hpf, and embryos are unlikely to survive after 96 hpf. SU5402 is considered to be a potent multitargeted receptor tyrosine kinase inhibitor that eliminates multisignaling pathways, thus leading to the development of severe malformations after 72 hpf and making the analysis of thyroid development difficult. Based on this, we restricted our analysis to PD166866 treatment alone. Treatment with an FGF inhibitor (PD166866) from 36 to 48 hpf caused a reduction in thyroid volume, thus indicating that treatment during this specific time window impairs thyroid budding. During the thyroid proliferation time window (after 48 hpf), drug‐induced vascular anomalies are the result of an indirect mechanism that causes abnormal positioning of the thyroid gland with little impact on thyroid volume (as shown in the 3D simulated images), and this results in a low impact on the number of thyroid cells. Previous studies have focused on FGF pathway inhibitor effects on thyroid budding in the early development of the thyroid (24–36 hpf); however, knowledge regarding its effects on thyroid expansion and proliferation is limited [17, 25]. In the current study, we focused on FGF pathway inhibitor effects on the later stages of thyroid development (36–96 hpf), and our results suggest that the FGF pathway inhibitor affects thyroid budding and the expansion period and not the proliferation period, as reflected by the thyroid volume in the 3D simulated images.

There are limitations to the 3D simulation method. As it depends on the fluorescence signal, stronger fluorescence results in greater ease of simulation creation. In our study, we obtained all photographic images using the same parameters and used embryos from the same batch in each experimental group. When simulating 3D images, the parameters were the same. It is advisable to compare the data from one batch of experiments.

## Conclusions

Three‐dimensional microscopy and image fusion reconstruction provided a comprehensive picture of the thyroid anatomy and complemented the anatomical fluorescence information. The FGF pathway inhibitor affects thyroid budding and expansion and does not affect proliferation.

## Conflict of interest

The authors declare no conflict of interest.

## Author contributions

MD and HDS conceptualized the study. MD designed methodology. RJZ provided software. LY, FS, and YF validated the data. FS and XPY involved in formal analysis. RJZ wrote the original manuscript and prepared the draft. MD wrote, reviewed, and edited the manuscript. MD visualized the data. HDS supervised the data; and MD and HDS acquired funding. All authors have read and agreed to the published version of the manuscript.

## Supporting information


**Video S1.** 3D simulated images of zebrafish thyroid at 48 hpf.

**Video S2.** 3D simulated images of zebrafish thyroid at 55 hpf.

**Video S3.** 3D simulated images of zebrafish thyroid at 72 hpf.

**Video S4.** 3D simulated images of zebrafish thyroid at 96 hpf.

**Video S5.** 3D simulated images of zebrafish thyroid at 120 hpf.

## Data Availability

The datasets used and/or analyzed during this study are available from the corresponding author on reasonable request.
